# Spondylodiscitis in Paediatric Patients: The Importance of Early Diagnosis and Prolonged Therapy

**DOI:** 10.3390/ijerph15061195

**Published:** 2018-06-07

**Authors:** Sonia Bianchini, Andrea Esposito, Nicola Principi, Susanna Esposito

**Affiliations:** 1 Pediatric Clinic, Department of Surgical and Biomedical Sciences, Università degli Studi di Perugia, 06123 Perugia, Italy; bianchini.sonia@fastwebnet.it; 2 Radiology Unit, Fondazione IRCCS Ca’ Granda Ospedale Maggiore Policlinico, 20122 Milan, Italy; rxandreaesposito@yahoo.it; 3 Università degli Studi di Milano, 20122 Milan, Italy; nicola.pricipi@unimi.it

**Keywords:** antibiotic therapy, magnetic resonance imaging, osteo-articular infections, osteomyelitis, spondylodiscitis

## Abstract

**Background:** Spondylodiscitis (SD), the concurrent infection of a vertebral disc and the adjacent vertebral bodies, is a very severe disease that can lead to death or cause spinal deformities, segmental instabilities, and chronic pain, which significantly reduces the quality of life for affected patients. Early diagnosis and treatment are essential in order to reduce the risk of negative outcomes. The two cases of SD that are described in this paper might be useful for informing paediatric approaches to children with SD. **Case presentation:** The cases that are reported here are about two children of approximately 2 and 3 years of age, in whom SD involving the L4–L5 and L3–L4 interspaces, had a subacute or chronic course. The clinical manifestations were mild, fever was absent, and the lumbar pain lasted for a long time and was the predominant symptom. Moreover, laboratory tests were in the normal range or only slightly abnormal, as were the standard radiographs of the lumbar spine. In both of the cases, SD confirmation was obtained through magnetic resonance imaging (MRI) and MRI was also used to evaluate the response to therapy. In both of our patients, tuberculosis was excluded based on tuberculin skin testing and the Quantiferon TBgold tests being negative. This finding led to the prescription of a broad-spectrum antibiotic therapy, including a drug that was potentially effective against Staphylococcus aureus (Sa). The strict monitoring of the spinal damage with MRI avoided the need for aspirations or biopsies; invasive procedures that are ethically acceptable in pediatric age only in a few selected cases, when the empirical antibiotic is associated with a worsening of spinal damage; or the vertebral osteomyelitis lesion mimics a tumoral lesion. **Conclusions:** Although rare, SD represents an important disease in children. In toddlers and preschool children, it can have a subacute or chronic course, in which only back pain, irritability, and walking difficulties are the signs and symptoms of the disease. MRI remains the best method for confirming the diagnosis and for evaluating therapy efficacy. Antibiotics are the drugs of choice. Although the duration has not been established, antibiotics should be administered for several weeks.

## 1. Background

Spondylodiscitis (SD), the concurrent infection of a vertebral disc and the adjacent vertebral bodies, is a very severe disease that can lead to death or cause spinal deformities and segmental instabilities, significantly reducing the quality of life for affected patients [1]. It is primarily known as a disease that affects adults and the elderly, as the estimated incidences in these subjects are 2.4 and 6.5 cases per 100,000, respectively. In contrast, in children, the incidence is no higher than 0.3 per 100,000 [2].

Early diagnosis and treatment are essential to reduce the risk of negative outcomes [3]. However, these goals are difficult to achieve in children, as the infrequency, together with the poor specificity of clinical manifestations and the inability of younger patients to verbalize symptoms, makes the diagnosis of paediatric SD very difficult. Moreover, treatment poses several problems. The aetiology can vary; the identification of the infecting organism requires invasive procedures, unless the blood culture is positive; and the empirical drug regimens are usually prescribed. Finally, the duration of treatment has not been established, although long-term drug administration is usually required to reduce symptoms and normalize laboratory tests [3]. The diagnosis and treatment of paediatric SD can be significantly delayed and this can lead to an increase in the risk of permanent spine abnormalities [4]. To reduce these problems, paediatricians should be alert and pay attention to the peculiarities of the clinical manifestations of SD at different ages, and should vary therapy according to the disease evaluation. The two cases of SD that are described in this paper might be useful for informing paediatric approaches to children with SD.

## 2. Case Presentations

### 2.1. Case 1

This case describes a 21 months old, previously healthy boy, who presented with weakness of the lower extremities and lumbar pain after a mild upper respiratory tract infection. For this reason, he was immediately brought to the paediatric emergency department where he underwent a hip ultrasound that excluded a joint effusion. Nevertheless, he was dismissed with a diagnosis of transient hips arthritis and was treated with anti-inflammatory therapy. A few days later, because of worsening pain and the inability to walk, he returned to our department and was hospitalized.

On admission, a spine radiograph showed a slight reduction in the thickness of the L5 soma. Moreover, a spine magnetic resonance imaging (MRI) showed the T1 post-enhancement increased signals of the anulus L4–L5, of the opposite end-plates of L4 and L5, of the adjacent soft tissues and of the osteolytic area of the L5 pedicle. Therefore, a diagnosis of SD with associated osteomyelitis was made.

The blood exams revealed an increase in the inerythrocyte sedimentation rate (ESR) (77 mm/h,) and C reactive protein (CRP) (2.17 mg/dL, normal values < 0.4 mg/dL). In contrast, the patient had a normal white blood cell count (WBC) count, a normal procalcitonin serum concentration (0.12 ng/mL, normal values < 0.25 ng/mL), and a negative Quantiferon TB-gold test.

Broad-spectrum intravenous therapy with meropenem (100 mg/kg/day in three doses) and vancomycin (40 mg/kg/day in three doses) was started. Anti-inflammatory treatment was used for the first week and stopped with the complete resolution of the child’s symptoms and his return to normal walking.

After 3 weeks of therapy, the patient developed leukopenia with severe neutropenia (lowest WBC value of 5410/mm^3^, with 80/mm^3^ neutrophils). As both of the administered drugs have been associated with neutropenia [5,6], therapy was withdrawn and replaced with ceftazidime (100 mg/kg/day in three doses), which was carried on for another week until the second MRI. The images from this exam, performed after 4 weeks of total therapy, showed a reduction in the enhanced contrast, although there was not a complete resolution of the inflamed and infected state.

**Figure 1:**
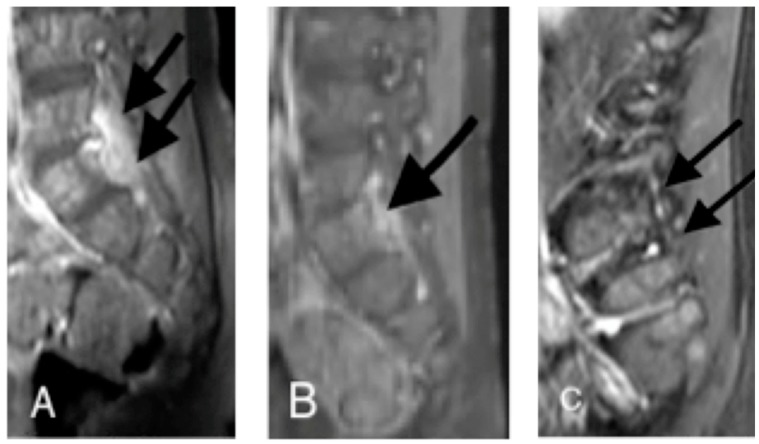
Lumbar spondylodiscitis with osteomyelitis, magnetic resonance imaging (MRI) of case 1. (**A**) Sagittal T1 post-enhancement image showing an increased signal of the annulus L4–L5, of the opposite end-plates of L4 and L5, of the adjacent soft tissues, and of an osteolytic area of the L5 pedicle (black arrow); (**B**,**C**) at the same level as A, after therapy (1 month and 4 months later, respectively), there is a progressive decrease in the pathological enhancement (black arrow), which has almost disappeared in C. Residual irregularities of the end-plates of L4 and L5 are evident.

Because of the radiological improvement, the normalization of the inflammatory factors, and the absence of symptoms in the child, he was discharged with an oral therapy of linezolid (30 mg/kg/day in three doses) and cefuroxime axetil (30 mg/kg/day in two doses). After 12 weeks of oral therapy, another MRI was performed. The images showed a complete resolution of the infectious process. Figure 1 shows differences between the MRI at admission and during the follow-up. Therefore, therapy was stopped (the patient received 16 weeks of therapy in total). The child was completely asymptomatic, and all of the blood exams, including the acute phase reactants and blood culture, were in the normal range or negative.

All of the blood exams that were performed to determine the nature of the infection did not show positivity for any recent, causative infectious agent. Additionally, the immunological and autoimmunity screenings were normal.

The 3 year follow up did not reveal any problem after discharge. The child never felt additional pain or had problems walking again.

### 2.2. Case 2

A 3 years old boy was admitted to our emergency department because he had been suffering from intermittent lumbar pain for several months and had difficulty walking for a few days. The patient’s personal medical history was uneventful until 4 months earlier when, playing with a friend, the child had a lumbar trauma that caused neither detectable skin lesions nor impairment to leg mobilization, and was not investigated. However, in the following weeks, the child started to feel pain whenever his father picked him up and was clearly more irritable than he had been in the past. A fever was never reported. Three months after the trauma, because of the increased lumbar pain, the child refused to walk. For this reason, he visited an emergency care unit and underwent a physical examination; laboratory blood tests, including a WBC and CRP serum level; and a full spine radiography. No abnormal results were detected. Oral therapy with a nonsteroidal anti-inflammatory drug for a week was prescribed. During this period, a partial resolution of the pain was demonstrated.

**Figure 2:**
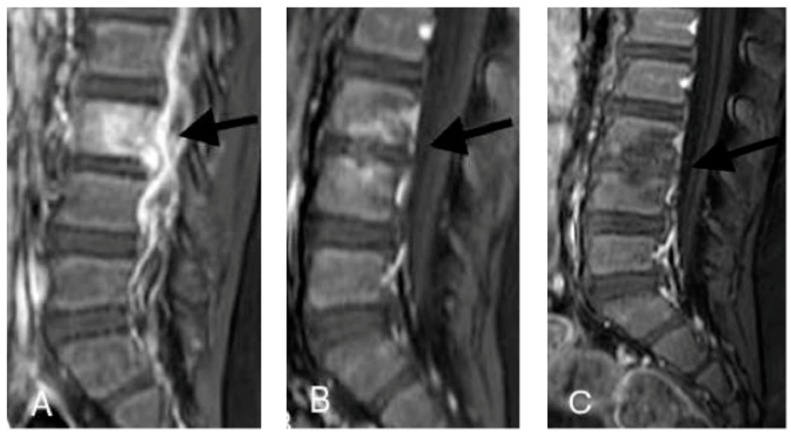
Lumbar spondylodiscitis, magnetic resonance (MRI) imaging of case 2. (**A**) Sagittal T1 post-enhancement image showing increased signal of the annulus L3–L4, of the opposite end-plates of L3 and L4, and of the adjacent soft tissues (black arrows); (**B**,**C**) at the same level as A, after therapy (1 month and 4 months later, respectively), there is a progressive decrease in the pathological enhancement (black arrows), which has almost disappeared in C. Residual irregularities of the end-plates of L3 and L4 are evident.

However, ten days after the drug discontinuation, the pain worsened. Therefore, the child was brought to our department. Here, a physical examination, an abdominal ultrasonography, and the laboratory blood tests were still normal or only slightly abnormal. The ESR reached 60 mm/h, CRP was 1.47 mg/dL (normal values < 0.4 mg/dL), and procalcitonin was 0.26 ng/mL (normal values < 0.25 ng/mL), but the patient had a normal WBC count. His body temperature was in the normal range. However, an MRI scan of the spine revealed that a T1 post-enhancement had increased the signal of the anulus L3–L4 of the adjacent soft tissue; this outcome is highly suggestive of an infective SD (Figure 2).

While awaiting the results of the blood culture, a broad-spectrum, anti-infective intravenous therapy was started with piperacillin–tazobactam (100 mg/kg/day divided into three doses) and vancomycin (40 mg/kg/day divided into three doses). Oral anti-inflammatory therapy was also provided.

The boy experienced rapid clinical improvement. In the first weeks, he stopped feeling pain and started walking again without lameness. Anti-inflammatory therapy was discontinued after several days. After 4 weeks of therapy, the boy underwent a second MRI, which showed no significant radiological change (Figure 2). The CRP was negative, but the ESR and procalcitonin were still slightly abnormal. The therapy was modified, and piperacillin-tazobactam was replaced by meropenem (100 mg/kg/day divided into three doses), while continuing vancomycin.

After 8 weeks of intravenous therapy, the inflammatory index was completely negative, and the child felt no more pain and could walk normally. The intravenous therapy was switched to oral therapy with linezolid (30 mg/kg/day in three doses) and cefuroxime axetil (30 mg/kg/day in two doses), and the child was discharged from the hospital.

After 12 weeks of total therapy, another MRI was performed to determine if therapy should be discontinued. The MRI showed an important reduction in the signal alterations, although residual irregularities of the end-plates of L3 and L4 were reported (Figure 2). On the basis of these findings, the child remained on antibiotic therapy for another month and then stopped. He received 16 weeks of therapy in total.

During hospitalization, all of the immunological exams that were performed were normal, and no causative infectious agent was documented. The whole therapy was well-tolerated, without any side effects.

During a 3 year follow-up, the child experienced only one episode of back pain, for which he promptly underwent an MRI that ruled out the possibility of a reactivation of the infection, but he showed a slightly adipose evolution of the L3 and L4 body. After that episode, which rapidly and spontaneously resolved, the child never felt lumbar pain again and maintained a normal life.

## 3. Discussion

The cases that were reported here were of two children of approximately 2 and 3 years of age, in whom SD involving the L4–L5 and L3–L4 interspaces had a subacute or chronic course. The clinical manifestations were mild, fever was absent, and lumbar pain lasted for a long time and was the predominant symptom. Moreover, the laboratory tests were in the normal range or only slightly abnormal, as were the standard radiographies of the lumbar tract of the spine. These findings were not surprising, as it had already been reported that the clinical manifestations of SD in children aged 6 months to 4 years were quite different from those that were usually evidenced in earlier and later periods of life [1,2,3]. In neonates and younger infants, SD was frequently a part of an acute clinical picture, in which severe sepsis with multiple organ failure was predominant. The disease is very severe, significant vertebral damage leading to kyphosis could occur, and neurological manifestations were common. In older children and adolescents, vertebral osteomyelitis was the most frequent sign, and patients had an acute disease with fever, appeared ill, and were in severe pain. Subacute, chronic manifestations were typical of SD in toddlers and preschool children [1,2,3]. Consequently, persistent lumbar pain and the emergence of abnormalities when walking, in a child of this age group, should lead paediatricians to include spinal infections among the diseases from which the patient might suffer. This focus seemed important when lumbar pain was accompanied by significant irritability, as was evidenced in the second of these cases. Kang et al. reported that approximately 60% of SD cases that were diagnosed in children <3 years showed that this symptom was the most common among all of the symptoms at the disease presentation [2].

When SD diagnosis was suspected, confirmation could be obtained through MRI. Bone scintigraphy with technetium (Tc-99m) had poor specificity. Positron emission tomography (PET) with 18 fluorodeoxyglucose had a distinctly higher spatial resolution, but the interpretation of a positive PET study was not easy and deserved cautions [7]. The most specific imaging method was MRI [8], which has currently been considered the as method of choice for the diagnosis of SD [9]. Together with the delayed diagnosis, a second problem complicated the final prognosis, which was confirmed in both of the reported cases, in which MRI was also used to evaluate the response to therapy. It had to be highlighted that the SD with the subacute or chronic course could be as a result of both pyogenic bacteria, as it commonly occurred in the first months of life, and Mycobacterium tuberculosis (Mt) [10,11]. This aetiology had to be attentively considered, because of the increased incidence of tuberculosis in industrialized countries and the emergence of multidrug resistant strains. In both of our patients, tuberculosis was excluded based on tuberculin skin testing and the Quantiferon TBgold test being negative. This finding led to the prescription of a broad-spectrum antibiotic therapy, including a drug that was potentially effective against Staphylococcus aureus (Sa). The choice of antibiotics was based on the knowledge that Sa was the most common aetiological agent of SD in all paediatric age groups, and that there were some negative rods that could cause the disease. As reported in the second case, the prescribed antibiotic therapy was not completely effective, as alterations of the vertebral bodies remained significant, even after several days of therapy. The strict monitoring of the spinal damage with MRI avoided the need for aspirations or biopsies; invasive procedures that were ethically acceptable in pediatric age only in a few selected cases, when the empirical antibiotic was associated with a worsening of spinal damage; or the vertebral osteomyelitis lesion mimics a tumoural lesion [12,13,14]. One open problem was the duration of therapy and which markers could suggest a cure and therefore no need for antibiotic administration. We used the MRI findings, but because of the expected lag behind the clinical finding, it was possible that they were not the best marker. Further studies in this regard are needed.

## 4. Conclusions

Although rare, SD represents an important disease in children. A rational approach favoring early diagnosis and appropriate treatment has to take into account that SD clinical manifestations vary according to age. In toddlers and preschool children, it can have a subacute or chronic course, in which only back pain, irritability, and walking difficulties are the signs and symptoms of the disease. MRI remains the best method to confirm the diagnosis and to evaluate therapy efficacy. Antibiotics are the drugs of choice, considering the role of Sa and of Mt. Duration has not been established, but, in accordance with what is currently recommended for osteomyelitis, antibiotics should be administered for several weeks. Final prognosis is generally good, especially when the therapy started early. However, an accurate monitoring of the response to therapy is needed, although the markers to suspend therapy are not defined.
